# Pre-donation assessment of cystatin C to improve prediction of pre- and post-donation GFR in potential living kidney donors

**DOI:** 10.1093/ndt/gfae065

**Published:** 2024-03-13

**Authors:** Jessica van der Weijden, Daan Kremer, Lisa B Westenberg, Jan-Stephan F Sanders, Robert A Pol, Ilja M Nolte, Martin H De Borst, Stefan P Berger, Stephan J L Bakker, Marco van Londen

**Affiliations:** Division of Nephrology, Department of Internal Medicine, University Medical Center Groningen, University of Groningen, Groningen, The Netherlands; Division of Nephrology, Department of Internal Medicine, University Medical Center Groningen, University of Groningen, Groningen, The Netherlands; Division of Nephrology, Department of Internal Medicine, University Medical Center Groningen, University of Groningen, Groningen, The Netherlands; Department of Surgery, University Medical Center Groningen, University of Groningen, Groningen, The Netherlands; Division of Nephrology, Department of Internal Medicine, University Medical Center Groningen, University of Groningen, Groningen, The Netherlands; Department of Surgery, University Medical Center Groningen, University of Groningen, Groningen, The Netherlands; Department of Epidemiology, University Medical Center Groningen, University of Groningen, Groningen, The Netherlands; Division of Nephrology, Department of Internal Medicine, University Medical Center Groningen, University of Groningen, Groningen, The Netherlands; Division of Nephrology, Department of Internal Medicine, University Medical Center Groningen, University of Groningen, Groningen, The Netherlands; Division of Nephrology, Department of Internal Medicine, University Medical Center Groningen, University of Groningen, Groningen, The Netherlands; Division of Nephrology, Department of Internal Medicine, University Medical Center Groningen, University of Groningen, Groningen, The Netherlands

**Keywords:** cystatin C, glomerular filtration rate, living kidney donation, muscle mass, post-donation GFR

## Abstract

**Background:**

Accurate estimation of glomerular filtration rate (GFR) is crucial in living kidney donation. While most estimated GFR (eGFR) equations are based on plasma creatinine, its levels are strongly influenced by muscle mass. Application of cystatin C (cysC)-based estimates before donation may improve both estimation of current GFR and prediction of post-donation GFR.

**Methods:**

We assessed the performance of Chronic Kidney Disease Epidemiology Collaboration equations based on creatinine (eGFR_creat-2009_, eGFR_creat-2021_), cysC (eGFR_CysC-2012_) or both (eGFR_combined-2012_, eGFR_combined-2021_) for estimating pre- and post-donation (mGFR) GFR in 486 living kidney donors. We subsequently focused on a subgroup of individuals with high/low muscle mass (25% highest/lowest 24-hour urinary creatinine excretion, sex stratified and height indexed).

**Results:**

Pre-donation eGFR_combined-2012_ and eGFR_combined-2021_ showed the strongest associations with pre- and post-donation mGFR. Pre-donation eGFR_combined-2021_ was most accurate for estimating both pre-donation (bias 0.01 ± 11.9 ml/min/1.73 m^2^) and post-donation mGFR (bias 1.3 ± 8.5 ml/min/1.73 m^2^). In donors with high/low muscle mass, cysC-based equations (with or without creatinine) performed better compared with equations based on only creatinine.

**Conclusions:**

Combined eGFR equations yielded a better estimate of pre- and post-donation mGFR compared with estimates based on creatinine or cysC only. The added value of cysC seems particularly pronounced in donors with high or low muscle mass.

KEY LEARNING POINTS **What was known:**

- Creatinine-based estimated glomerular filtration rate (eGFR) is inaccurate in potential living kidney donors due to underestimation and influences of muscle mass.
- Cystatin C (cysC) is less influenced by muscle mass and has been shown to improve the estimation of GFR when added to creatinine-based eGFR equations [Chronic Kidney Disease Epidemiology Collaboration (CKD-EPI)] in cross-sectional analyses in potential kidney donors.

**This study adds:**

- This study shows that the CKD-EPI equations that include both creatinine and cysC, when applied at pre-donation, also improve prediction of the 3-month post-donation GFR.
- The improvement was particularly pronounced in a subgroup of donors with high/low muscle mass (25% lowest and 25% highest height-indexed 24-hour creatinine excretion).

**Potential impact:**

- Accurate assessment of pre-donation GFR as well as prediction of post-donation GFR is highly important for living kidney donor evaluation.
- Estimating pre-donation GFR based on both creatinine and cystatin C could improve donor risk assessment and may therefore contribute to more informed decision making.

## INTRODUCTION

Assessment of kidney function plays an important role in the evaluation of potential living kidney donors, mainly to determine whether both the donor and the recipient will have sufficient kidney function after the donation or transplantation, respectively [1, 2]. So far, there has been no consensus—and thus no uniform policy—on how to assess pre-donation glomerular filtration rate (GFR) in potential donors [1, 3–5]. While determining GFR using exogenous filtration markers [measured GFR (mGFR)] is the gold standard, it is not widely implemented due to financial and practical constraints. Estimating GFR using plasma creatinine–based estimation equations is easier and less costly, but drawbacks of this method include inaccuracy due to influences of non-GFR determinants, such as muscle mass [6, 7]. Moreover, previous studies concluded that relying on estimated GFR (eGFR) for the selection of living kidney donors results in unjustified exclusion of donors due to imprecision of eGFR equations [6, 8–13]. While it is important to accurately estimate pre-donation GFR in potential donors at the time of evaluation, the goal is to assess whether sufficient kidney function will remain after donation.

We recently developed an equation based on pre-donation plasma creatinine, age and sex to predict post-donation mGFR in living kidney donors [14]. While the new equation outperformed plasma creatinine–based eGFR [Chronic Kidney Disease Epidemiology Collaboration (CKD-EPI) 2009], it still explained <40% of the variation in post-donation mGFR. In addition, this equation might perform worse in potential donors with muscle mass that deviates from average.

In recent decades, plasma cystatin C (cysC) has been proposed as a promising marker to estimate GFR, as it is less dependent on body size and composition [7]. Non-GFR determinants of plasma cysC include inflammation, diabetes and thyroid dysfunction [15–18], all of which are generally absent in potential living kidney donors. Addition of cysC improved the accuracy and precision of the CKD-EPI equations, which has been confirmed in living kidney donors in cross-sectional analyses, both pre- and post-donation [19–22], yet its added value in pre-donation prediction of post-donation GFR remains unclear. Therefore, we investigated whether pre-donation addition of plasma cysC to creatinine-based eGFR equations improves the prediction of both pre- and post-donation GFR in a prospective cohort of living kidney donors with available data on iothalamate-measured GFR. We specifically investigated the added value of cysC in donors with low or high muscle mass.

## MATERIALS AND METHODS

### Study design and population

**Figure 1:**
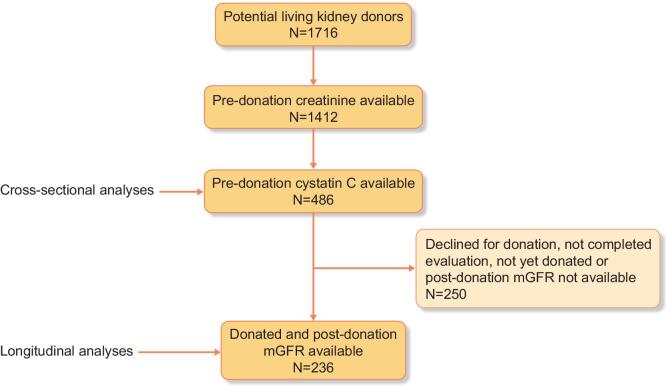
Overview of the study population.

For this study, we used data from the ongoing, prospective TransplantLines: The Transplantation Biobank study (ClinicalTrials.gov identifier: NCT03272841), which aims to assess short- and long-term outcomes after solid organ transplantation and donation [23]. For the current study, we selected 486 (potential) kidney donors enrolled in the TransplantLines study, with available pre-donation plasma creatinine and plasma cysC (Fig. 1). In 236 donors, the 3-month post-donation mGFR was available. Pre-donation mGFR was missing in six cases. All patients were evaluated for donation between 2016 and 2021 at the University Medical Center Groningen in Groningen, The Netherlands. The study was approved by the institutional ethical review board (METc 2014/077). All procedures were conducted in accordance with the Declaration of Helsinki and Declaration of Istanbul.

### Measurement of plasma creatinine, plasma cysC, eGFR and mGFR

Plasma cysC concentrations were measured in ethylenediaminetetraacetic acid plasma using validated particle-enhanced turbidimetric immunoassays (Gentian, Moss, Norway, for 198 patients and Roche Diagnostics, Basel, Switzerland for 38 patients), which are both calibrated assays. Plasma creatinine was measured routinely in our central chemistry laboratory by an isotope dilution mass spectrometry (IDMS) traceable enzymatic assay on the Roche Modular (Roche Diagnostics). The eGFR was calculated according to CKD-EPI equations based on plasma creatinine (eGFR_creat-2009_ and eGFR_creat-2021_), cysC (eGFR_cysC-2012_) and based on both markers combined (eGFR_combined-2012_ and eGFR_combined-2021_) as well as according to the creatinine-based and cysC-based European Kidney Function Consortium (EKFC) equations [7, 24–27]. Since all included donors were white, no correction for race was applied.

The mGFR was determined using ^125^I-iothalamate and ^131^I-hippurate infusion, as previously described [6]. In short, after drawing a blood sample, ^125^I-iothalamate and ^131^I-hippurate infusions were started (0.04 ml/kg containing 0.04 MBq and 0.03 MBq, respectively). At 8:00 a.m., 0.6 MBq of ^125^I-iothalamate was administered, followed by continuous infusion of 12 ml/h. After a 2-h stabilization period, baseline measurements were performed in a steady state of plasma tracer levels. Clearances were calculated as (U*V)/*P* and (I*V)/*P*, where U*V represents the urinary excretion, I*V represents the infusion rate of the tracer and *P* represents the plasma tracer concentration per clearance period. We calculated the mGFR from clearance levels of these tracers using (U*V)/*P* and corrected the renal clearance of ^125^I-iothalamate for urine collection errors by multiplying the urinary ^125^I-iothalamate clearances by the ratio of plasma and urinary ^131^I-hippurate clearance.

### Statistical analyses

In primary analyses, we investigated the performance of the pre-donation CKD-EPI equations to predict pre- and post-donation mGFR. This was done by univariable linear regression analysis and by assessment of accuracy and precision. Accuracy and precision were determined by the *R*^2^, bias, root mean squared error (RMSE), interquartile range of the bias and the percentage of eGFR values that differed <30% and <10% from the mGFR (*P*_30_ and *P*_10_, respectively). For cross-sectional accuracy (486 donors), we calculated the difference between pre-donation eGFR and pre-donation mGFR. For longitudinal accuracy (236 donors), we calculated the predicted post-donation mGFR by multiplying the pre-donation eGFR by 0.66, which was based on the mean change in pre- to post-donation mGFR in our cohort (−34%). This change is in line with current literature on short-term compensation of the remaining kidney [28]. The bias was then calculated as the difference between the predicted value of post-donation mGFR (0.66*pre-donation eGFR) and the true mGFR. We visualized the capacity of each equation to discriminate whether pre-donation GFR is greater than or less than 90 or 60 ml/min/1.73 m^2^ in scatter plots, as previously done by Gaillard *et al.* [13].

In secondary analyses, we investigated the association of pre-donation plasma creatinine and cysC with pre- and post-donation mGFR in uni- and multivariable linear regression analyses while adjusting for age and sex. The main reason to do so was to investigate whether our previously developed prediction equation for post-donation mGFR based on pre-donation creatinine, age and sex could be improved by the addition of cysC [14]. Next, we selected a subgroup of donors with low or high muscle mass by calculating the 24-h urinary creatinine excretion as a surrogate marker for muscle mass, which was indexed for height (missing in 36 donors who were not included in the quartiles) [29]. Donors were assigned to the subgroup (*n* = 243 of 486 for the cross-sectional analyses and *N* = 118 of 236 for the longitudinal analyses) if they were in the lowest (cross-sectional *N* = 122, longitudinal *N* = 59) or highest quartile (cross-sectional *n* = 122, longitudinal *n* = 59) of height-indexed 24-h creatinine excretion, stratified for sex. We repeated the univariable linear regression analyses of pre-donation CKD-EPI equations, plasma creatinine and cysC in this subgroup. In sensitivity analyses, we repeated the univariable linear regression analyses in subgroups according to the cysC assay and in subgroups stratified for sex. Statistical analyses were performed in SPSS version 23 for Windows (IBM, Armonk, NY, USA), R version 3.0.1 (R Foundation for Statistical Computing, Vienna, Austria) and GraphPad Prism 8 for Windows (GraphPad Software, San Diego, CA, USA). *P*-values <.05 were considered statistically significant.

## RESULTS

### Characteristics of the living kidney donor population

Characteristics of the living kidney donor population are shown in Table 1. At pre-donation, age was 56 ± 11 years, 54% of the donors were female and the body mass index (BMI) was 26 ± 5 kg/m^2^.

**Table 1:** Pre-donation characteristics of the total living kidney donor population and the subgroup with post-donation mGFR available

	Pre-donation		Post-donation
Characteristics	Total cohort	Subgroup with post-donation mGFR available	Subgroup with post-donation mGFR available

Population, *n*	486	236	236
Age (years), mean ± SD	56 ± 11	56 ± 11	57 ± 11
Female, *n* (%)	261 (54)	120 (51)	120 (51)
Weight (kg)	80 ± 13	80 ± 13	79 ± 13
Height (cm)	174 ± 9	175 ± 9	174 ± 9
BMI (kg/m^2^)	26 ± 5	26 ± 4	26 ± 3
BSA (m^2^)	1.94 ± 0.19	1.95 ± 0.19	1.94 ± 0.18
Waist:hip ratio	0.90 ± 0.10	0.90 ± 0.10	0.91 ± 0.10
SBP (mmHg)	126 ± 14	126 ± 14	125 ± 12
Plasma creatinine (µmol/l)	77 ± 14	77 ± 14	–
Plasma cysC (mg/l)	0.89 ± 0.15	0.86 ± 0.14	–
mGFR (ml/min/1.73 m^2^)	94 ± 16	96 ± 14	62 ± 10
eGFR_creat-2009_ (ml/min/1.73 m^2^)	87 ± 15	89 ± 14	–
eGFR_cysC-2012_ (ml/min/1.73 m^2^)	90 ± 17	94 ± 16	–
eGFR_combined-2012_ (ml/min/1.73 m^2^)	90 ± 15	93 ± 14	–
eGFR_creat-2021_ (ml/min/1.73 m^2^)	90 ± 14	91 ± 13	–
eGFR_combined-2021_ (ml/min/1.73 m^2^)	94 ± 14	96 ± 14	–
EKFC_creat_ (ml/min/1.73 m^2^)	82 ± 14	83 ± 13	–
EKFC_cysC_ (ml/min/1.73 m^2^)	83 ± 15	86 ± 14	–
EKFC_combined_ (ml/min/1.73 m^2^)	83 ± 13	84 ± 12	–

Values are presented as mean ± standard deviation unless stated otherwise.

BSA: body surface area; SBP: systolic blood pressure.

### Primary analyses

#### Prediction of pre-donation mGFR with pre-donation eGFR

**Figure 2:**
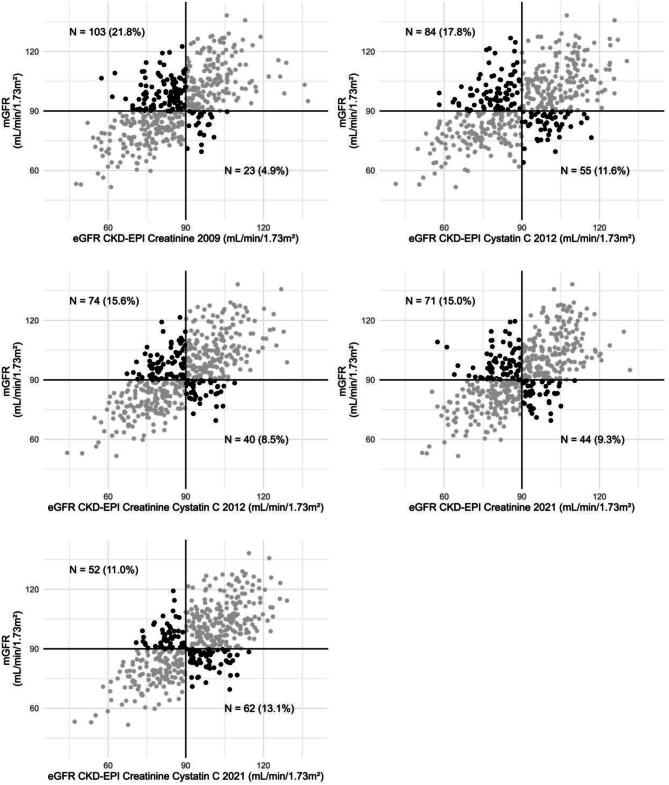

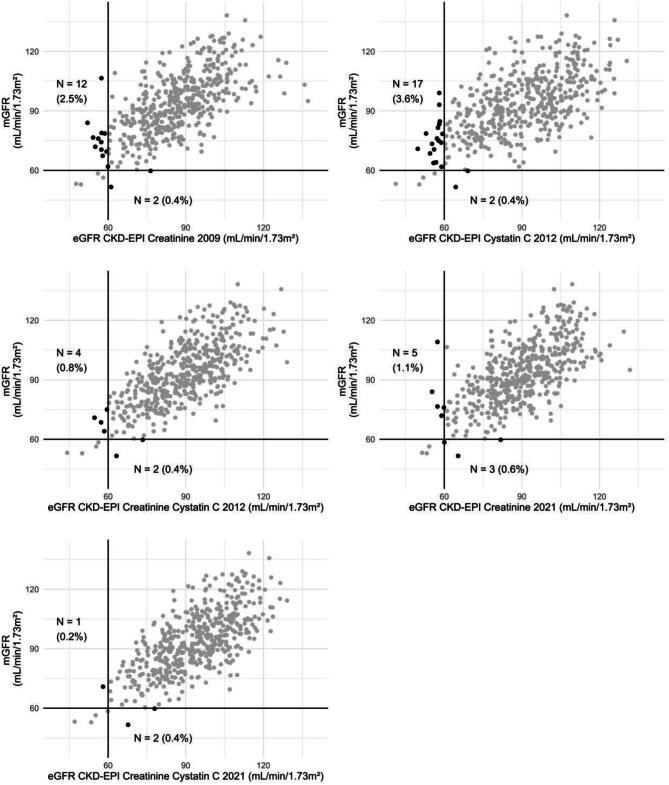
Capacity of the CKD-EPI equations to discriminate between pre-donation eGFR greater than or less than 90 and 60 ml/min/1.73 m^2^.

The eGFR_combined-2012_ and eGFR_combined-2021_ equations had the highest standardized β (Sβ) for the association with pre-donation mGFR [0.70 (95% CI 0.64–0.77) and 0.69 (95% CI 0.62–0.75), respectively; Table 2]. When looking at the predictive capacity of pre-donation eGFR for pre-donation mGFR (Table 3), the bias of the eGFR_creat-2009_ was −6.7 ml/min/1.73 m^2^ (95% CI −8.0–5.7), with an RMSE of 12.7 ml/min/1.73 m^2^. For eGFR_creat-2009_, the *P*_30_ was 97% (95% CI 95–98) and the *P*_10_ was 49% (95% CI 45–54). Combining creatinine with cysC in the eGFR_combined-2012_ resulted in a bias of −4.2 ml/min/1.73 m^2^ (95% CI −5.3–3.1), an RMSE of 11.7 ml/min/1.73 m^2^ and a *P*_30_ and *P*_10_ of 99% (95% CI 98–99.6) and 55% (95% CI 51–60), respectively. The update of the eGFR_combined-2012_ to the eGFR_combined-2021_ resulted in the lowest bias of all equations [0.01 ml/min/1.73 m^2^ (95% CI −1.1–1.1)] with a comparable RMSE, interquartile range of the bias, *P*_30_ and *P*_10_. The EKFC equations were less accurate and less precise compared with the CKD-EPI equations that included both creatinine and cysC. Bland–Altman plots of the eGFR_creat-2009_, eGFR_cysC-2012_ and eGFR_combined-2021_ are shown in Supplementary Figure S1. The capacity of each eGFR equation to discriminate pre-donation GFR greater than or less than 90 and 60 ml/min/1.73 m^2^ is shown in Fig. 2.

**Table 2:** Univariable linear regression analyses of pre-donation plasma creatinine/cysC and clinical characteristics with pre- and post-donation mGFR

	Pre-donation					
	Total cohort (*N* = 486)			Muscle mass subgroup (*n* = 243)		
	Sβ (95% CI)	*R* ^2^	*P-*value	Sβ (95% CI)	*R* ^2^	*P*-value
mGFR	–	–	–	–	–	–
EKFC_combined_	0.71 (0.65–0.77)	0.51	<.001	0.73 (0.64–0.82)	0.53	<.001
EKFC_creat_	0.68 (0.61–0.74)	0.46	<.001	0.64 (0.55–0.74)	0.41	<.001
EKFC_cysC_	0.60 (0.52–0.67)	0.36	<.001	0.65 (0.56–0.75)	0.42	<.001
eGFR_combined-2021_	0.69 (0.62–0.75)	0.47	<.001	0.69 (0.60–0.79)	0.48	<.001
eGFR_creat-2021_	0.64 (0.57–0.71)	0.41	<.001	0.59 (0.48–0.69)	0.34	<.001
eGFR_combined-2012_	0.70 (0.64–0.77)	0.49	<.001	0.71 (0.62–0.80)	0.50	<.001
eGFR_cysC-2012_	0.60 (0.52–0.67)	0.36	<.001	0.63 (0.54–0.73)	0.40	<.001
eGFR_creat-2009_	0.65 (0.58–0.72)	0.42	<.001	0.61 (0.50–0.71)	0.36	<.001
Plasma cysC	−0.50 (−0.58 to −0.42)	0.25	<.001	−0.53 (−0.64 to −0.43)	0.28	<.001
Plasma creatinine	−0.31 (−0.40 to −0.22)	0.09	<.001	−0.20 (−0.33 to −0.08)	0.04	.002
	**Post-donation**					
	**Total cohort (*N* = 236)**			**Muscle mass subgroup (*n* = 118)**		
	**Sβ (95% CI)**	***R* ^2^**	***P*-value**	**Sβ (95% CI]**	***R* ^2^**	***P*-value**

mGFR	0.75 (0.67–0.84)	0.58	<.001	0.80 (0.69–0.92)	0.59	<.001
EKFC_combined_	0.63 (0.53–0.73)	0.40	<.001	0.66 (0.52–0.80)	0.43	<.001
EKFC_creat_	0.56 (0.45–0.66)	0.31	<.001	0.52 (0.37–0.68)	0.27	<.001
EKFC_cysC_	0.56 (0.45–0.66)	0.31	<.001	0.63 (0.48–0.77)	0.39	<.001
eGFR_combined-2021_	0.60 (0.50–0.71)	0.36	<.001	0.63 (0.48–0.77)	0.38	<.001
eGFR_creat-2021_	0.52 (0.41–0.63)	0.27	<.001	0.47 (0.31–0.63)	0.22	<.001
eGFR_combined-2012_	0.63 (0.53–0.73)	0.39	<.001	0.65 (0.51–0.79)	0.40	<.001
eGFR_cysC-2012_	0.53 (0.43–0.64)	0.28	<.001	0.60 (0.46–0.75)	0.34	<.001
eGFR_creat-2009_	0.56 (0.45–0.66)	0.31	<.001	0.51 (0.36–0.67)	0.25	<.001
Plasma cystatin C	−0.46 (−0.58 to −0.35)	0.21	<.001	−0.54 (−0.70 to −0.39)	0.27	<.001
Plasma creatinine	−0.31 (−0.44 to −0.19)	0.09	<.001	−0.24 (−0.41 to −0.06)	0.05	.01

**Table 3:** Accuracy and precision of the eGFR equations for pre- and post-donation mGFR.

Variables	eGFR_creat-2009_	eGFR_cysC-2012_	eGFR_combined-2012_	eGFR_creat-2021_	eGFR_combined-2021_	EKFC_creat_	EKFC_cysC_	EKFC_combined_
	Accuracy and precision pre-donation eGFR for pre-donation mGFR^a^							
*R* ^2^	0.42	0.36	0.49	0.41	0.47	0.46	0.36	0.51
Bias (95% CI)	−6.7 (−8.0 to −5.7)	−3.5 (4.8 to −2.1)	−4.2 (−5.3 to −3.1)	−3.5 (−4.6 to −2.3)	0.01 (−1.1–1.1)	−11.4 (−12.5 to −10.3)	−10.6 (−11.9 to −9.4)	−11.0 (−12.0 to −10.0)
RMSE	12.7	14.8	11.7	12.6	11.9	12.0	13.9	11.1
IQR bias	−15.1–0.9	−13.7–7.5	−12.7–3.7	−12.1–4.6	−8.5–8.5	−19.2–3.4	−20.5 to −1.5	−19.0 to −3.6
*P*_30_ (95% CI)	96.6 (94.5–97.9)	94.1 (91.5–95.9)	98.9 (97.5–99.6)	97.5 (95.6–98.6)	97.9 (96.1–98.9)	94.9 (932.5–96.6)	91.5 (88.7–93.8)	98.1 (96.5–99.1)
*P*_10_ (95% CI)	49.0 (44.6–53.5)	44.4 (40.0–58.9)	55.2 (50.7–59.6)	54.3 (49.8–58.8)	54.5 (50.0–59.0)	38.7 (34.4–43.2)	37.4 (33.2–41.9)	38.9 (34.6–43.4)
	Accuracy and precision pre-donation eGFR for post-donation mGFR^b^							
*R* ^2^	0.31	0.28	0.39	0.27	0.36	0.31	0.31	0.40
Bias (95% CI)	−3.5 (−4.7 to −2.4)	−0.5 (−1.8–0.8)	−1.2 (−2.3 to −0.2)	−2.0 (−3.2 to −0.9)	1.3 (0.2–2.3)	−7.3 (−8.5 to −6.2)	−5.8 (−7.0 to −4.7)	−6.6 (−7.6 to −5.6)
RMSE	9.0	10.0	8.2	9.2	8.5	8.7	9.1	7.8
IQR bias	−9.1–2.1	−7.0–5.2	−6.1–4.1	−8.2–4.1	−4.0–6.6	−13.2–1.2	−11.6 to −0.3	−11.7 to −1.8
*P*_30_ (95% CI)	95.3 (91.8–97.5)	92.8 (88.7–95.5)	96.6 (93.3–98.4)	96.6 (93.3–98.4)	96.2 (92.8–98.1)	93.2 (89.2–95.9)	92.8 (88.7–95.5)	96.6 (93.3–98.4)
*P*_10_ (95% CI)	50.8 (44.5–57.2)	48.7 (42.4–55.1)	61.0 (54.7–67.0)	49.6 (43.3–55.9)	53.4 (47.0–59.6)	39.8 (33.8–46.2)	40.3 (34.2–46.6)	44.9 (38.7–51.3)

a Bias calculated as eGFR − mGFR: positive bias represents overestimation and negative bias represents underestimation.

b For calculation of the bias of pre-donation eGFR for post-donation mGFR, we first calculated the predicted post-donation mGFR value by multiplying pre-donation eGFR by 0.66. The bias was then calculated as the difference between predicted post-donation mGFR (0.66*pre-donation eGFR) and true mGFR: positive bias represents overestimation and negative bias represents underestimation.

IQR: interquartile range; *P*_30_ and *P*_10_: percentage of bias within 30% or 10% of mGFR.

#### Prediction of post-donation mGFR with pre-donation eGFR

Consistent with the cross-sectional analyses, the eGFR_combined-2012_ and eGFR_combined-2021_ equations had the highest Sβ [0.63 (95% CI 53–73) and 0.60 (95% CI 0.50–0.71), respectively; Table 2 and Supplementary Figure S2] for the association with post-donation mGFR (*n* = 236). For the prediction of post-donation mGFR (Table 3), the bias was calculated as (pre-donation eGFR*0.66) − true post-donation mGFR. The eGFR_creat-2009_ equation had a bias of −3.5 ml/min/1.73 m^2^ (95% CI −3.7–2.3) and an RMSE of 9.0 ml/min/1.73 m^2^. The combined eGFR_combined-2012_ equation had a bias of −1.2 ml/min/1.73 m^2^ (95% CI −2.3–0.2) and an RMSE of 8.2 ml/min/1.73 m^2^, comparable to the eGFR_creat-2021_ equation. Although the eGFR_cysC-2012_ equation seemed to have the lowest bias, this equation had the lowest *P*_30_ and *P*_10_ [93% (95% CI 89–96) and 49% (95% CI 42–45), respectively]. The eGFR_combined-2012_ and eGFR_combined-2021_ equations had the highest *P*_30_ and *P*_10_. Again, the EKFC performed worse than the CKD-EPI equations including creatinine and cysC.

### Secondary analyses

#### Associations of pre-donation plasma cysC and creatinine with pre- and post-donation mGFR

Pre-donation plasma cysC showed a stronger association with pre-donation mGFR than plasma creatinine [cysC: Sβ = −0.50 (95% CI −0.58–0.42); creatinine: Sβ = −0.31 (95% CI −0.40–0.22); Table 2]. Similar differences were observed for the association of pre-donation cysC and creatinine with post-donation mGFR. The addition of pre-donation cysC to a multivariable linear regression model containing pre-donation creatinine, age and sex predicting pre-donation mGFR significantly improved the model *R*^2^ from 0.47 to 0.53 (*P* < .001, Supplementary Table S1). For post-donation mGFR, the *R*^2^ increased significantly from 0.32 to 0.40 (*P* < .001; Supplementary Table S2).

#### Donors with high or low muscle mass

We defined a subgroup that included donors with muscle mass in the lowest and highest quartiles based on height-indexed 24-h creatinine excretion to study whether estimation of GFR improves with cysC in a group in whom plasma creatinine concentrations might be affected by muscle mass (characteristics in Supplementary Table S3). The two quartiles were combined, after which the univariable linear regression analyses were repeated (Table 2). In this subgroup, the strength of the association of creatinine (normally distributed) with both pre- and post-donation mGFR decreased [Sβ = −0.20 (95% CI −0.33–0.08), *P* = .002 (pre-donation mGFR)], whereas the strength of the association between cysC and post-donation mGFR increased [Sβ = −0.53 (95% CI −0.64–0.43), *P* < .001 (pre-donation mGFR)]. The eGFR_creat-2009_ and eGFR_creat-2021_ equations showed the weakest correlations with pre- and post-donation mGFR, and also in this subgroup, the eGFR_combined-2012_ and eGFR_combined-2021_ equations showed the strongest association with pre- and post-donation mGFR. Bland–Altman plots of cross-sectional performance of the eGFR_creat-2009_, eGFR_cysC-2012_ and eGFR_combined-2021_ equations are shown in Supplementary Figure S3. Scatter plots of the longitudinal performance of the eGFR_creat-2009_, eGFR_cysC-2012_ and eGFR_combined-2021_ equations are shown in Supplementary Figure S4.

### Sensitivity analyses

We performed a sensitivity analysis where we stratified the cohort according to the cysC assay that was used (Roche, *n* = 146; Gentian, *n* = 40). Repetition of the univariable linear regression analyses in these subgroups yielded similar results (Supplementary Tables S4 and S5). Similarly, repetition of analyses stratified for sex did not change the results of this study (Supplementary Tables S6–S9).

## DISCUSSION

This study aimed to investigate whether pre-donation cysC-based (with or without creatinine) GFR estimation could improve assessment of pre- and post-donation GFR in living kidney donors. We found that the eGFR_combined-2012_ and eGFR_combined-2021_ equations showed stronger associations with pre- and post-donation mGFR than the CKD-EPI equations based on either creatinine or cysC alone. The pre-donation eGFR_combined-2012_ and eGFR_combined-2021_ equations were also most accurate and precise for pre- and post-donation mGFR. The addition of cysC to a multivariable linear regression model containing age, sex and plasma creatinine significantly increased the explained variance in pre- and post-donation mGFR. Improvements in associations with pre- and post-donation mGFR when cysC was used for pre-donation GFR estimation were particularly pronounced in subgroups of donors with high and low muscle mass. Our study supports the added value of pre-donation cysC as a marker of pre- and post-donation kidney function in potential living kidney donors.

The Kidney Disease: Improving Global Outcomes Living Kidney Donor Guideline (2017) recommends confirming GFR using one or more of the following methods: mGFR, measured creatinine clearance, eGFR (eGFR_combined-2012_) and/or repeated eGFR_creat-2009_ [5]. All these methods are different in terms of costs, feasibility and availability and also in terms of accuracy and precision, and therefore more clear guidance is needed. In recent decades, cysC has emerged as a promising marker of kidney function, being less dependent on body size and composition than creatinine [15]. Th addition of cysC to the CKD-EPI equations has been shown to improve accuracy and precision in cross-sectional analyses [7, 19, 25, 30, 31]. Additionally, it has been shown recently that combining creatinine and cysC improves the accuracy and precision of the EKFC equation [32]. Our study showed that the addition of pre-donation cysC to a pre-donation creatinine-based multivariable linear regression model that was used to develop a prediction equation in a previous study by our group [14] improved the model fit for both pre- and post-donation mGFR. The pre-donation eGFR_combined-2012_ and eGFR_combined-2021_ equations showed stronger associations with pre- and post-donation mGFR than the CKD-EPI equations that only included creatinine or cysC. In addition, the pre-donation eGFR_combined-2012_ and eGFR_combined-2021_ showed better accuracy and precision for predicting pre- and post-donation mGFR. Future studies should investigate whether prediction of post-donation mGFR can be improved with donor-specific cysC-based (with or without creatinine) donor equations or whether the existing CKD-EPI equations are sufficient.

Due to the effects of muscle mass on plasma creatinine concentrations, plasma creatinine–based GFR assessment might not be accurate in individuals with muscle mass that deviates from average (i.e. the population the model was based on). Our subgroup analyses confirm these concerns. In donors with high or low muscle mass, the association of pre-donation creatinine with pre- and post-donation mGFR decreases, while pre-donation cysC strongly associates with pre- and post-donation mGFR in this subgroup. This indicates that influences of muscle mass on serum creatinine concentrations may not only be problematic in the extremes of muscle mass, but also within the normal ranges, since the rate of creatinine excretion in our population was highly comparable to the Swiss cross-sectional study [33]. This translates into stronger associations of the CKD-EPI equations that include cysC than the creatinine-based CKD-EPI equations with pre- and post-donation mGFR. This is in line with prior studies stating that the ratio between creatinine and cysC is a useful predictor for sarcopenia [34–36]. While many studies have concluded that there is no association between cysC concentrations and muscle mass [37–39], Ivey-Miranda *et al.* [40] found a significant association between muscle mass (assessed by creatinine excretion) and cysC in heart failure patients. However, as stated by the authors, the association was less strong than the association of creatinine with muscle mass and might be secondary to non-GFR determinants of cysC in this unhealthy population [40]. Similarly, Macdonald *et al.* [41] found a correlation between lean body mass and cysC, after adjusting for GFR, which they deemed logical since cysC is produced by all nucleated cells in the body, including muscle cells. Therefore cysC might not be totally independent of muscle mass, but since it is not only produced by muscle cells, it might be superior to creatinine in patients with high or low muscle mass, which is supported by the results of our study.

Other non-GFR determinants of plasma cysC that have been described include inflammation, thyroid dysfunction, diabetes, C-reactive protein, white blood cell count and plasma albumin concentration [15, 18], but the exact pathways through which these affect plasma concentrations of cysC are not fully understood. Other studies show that there is no direct relation between inflammation and plasma cysC concentrations [42, 43]. Thus it is not clear when cysC-based eGFR should be interpreted with caution. It could be that these determinants are less variable or even absent in healthy individuals, making this a promising marker of kidney function in potential kidney donors, as was shown previously [44]. Additionally, cysC has a greater molecular weight (13 kDa) than creatinine (0.113 kDa), which may result in an earlier decrease in filtration of cysC in diseases that affect the glomerular filtration barrier [45, 46].

We found two prior studies that investigated the longitudinal association of cysC with the change in GFR from pre- to post-donation [47, 48]. Both studies found no advantages of cysC compared with plasma creatinine, but they were relatively small and did not use mGFR as a reference method. In 2017, Bang *et al.* [49] showed that pre-donation plasma cysC is a better marker of kidney function recovery after living kidney donation than eGFR determined by the Modification of Diet in Renal Disease equation. To the best of our knowledge, our study is the first to investigate the performance of pre-donation plasma cysC and the cysC-based CKD-EPI equations to assess absolute post-donation mGFR.

Guidelines agree that relying on creatinine-based GFR assessment for selection of potential donors is insufficient for final decision making [5, 50]. There is a group of donors in which post-donation mGFR is low despite high pre-donation creatinine-based eGFR [14] but, at the same time, it has been shown that relying on plasma creatinine–based GFR assessment could lead to needless exclusion of potential donors [13, 51]. Our data show that cysC-based eGFR may be more accurate, but not more (or even less) precise than creatinine-based eGFR. Because imprecision of an equation may be problematic at the individual donor level, we do not suggest replacing creatinine-based eGFR with cysC-based eGFR for living kidney donor selection. Our study shows that estimation of GFR improves when based on the combination of plasma creatinine and cysC and therefore we recommend measuring both in potential donor candidates. Comparable performance of the eGFR_combined-2021_ and eGFR_combined-2012_ equations was in line with findings of Inker *et al.* [25] in non-black individuals in the development study of the CKD-EPI 2021 equation. These results and the ethical concerns about the prior CKD-EPI equations may favour use of the race-free eGFR_combined-2021_ equation. We hypothesize that the eGFR_combined-2021_ also performs better than the eGFR_combined-2021_ in non-white donors, since performance also improved in black individuals in the general population, with smaller differential bias compared with non-black individuals, but performance in other populations remains to be investigated [25]. Also, the precision and accuracy of cysC and/or creatinine-based equations was never high enough to abandon mGFR. Therefore, when available, mGFR is still preferred. If doubt exists whether pre-donation GFR is sufficient, we suggest referral to a transplant centre that has mGFR available.

The strengths of this study include the availability of both plasma creatinine and cysC as well as mGFR. Additionally, inclusion of declined donors in cross-sectional analyses reduced the risk of selection bias. Moreover, the availability of data on muscle mass enabled us to investigate the performance of cysC in donors with poor performance of creatinine. Yet the study also has limitations. First, the relatively small sample size and the single-centre design of the study hampered investigating the predictive capacity (accuracy and precision) of the CKD-EPI equations in internal and external validation data sets. Second, our study consisted of only white donors, which may impact the performance of the CKD-EPI equations [7, 24, 25]. Third, the longitudinal analyses, by definition, only included donors who were accepted for donation, possibly introducing selection bias. Lastly, although our index of muscle mass, i.e. height-indexed 24-h creatinine excretion, is a surrogate marker of total muscle mass [29], it may itself be affected by GFR, tubular secretion or protein intake.

In conclusion, our data suggest that pre-donation GFR estimation based on the combination of creatinine and cysC improves the prediction of pre- and post-donation mGFR compared with GFR estimation based on either of these markers alone. The added prognostic value of cysC seemed particularly pronounced in donors with high or low muscle mass.

## Supplementary Material

gfae065_Supplemental_File

## Data Availability

The data that support the findings of this study are available upon reasonable request from the corresponding author. The data are not publicly available due to the privacy of the research participants.
