# Supplementary material for: Pre-donation assessment of cystatin C to improve prediction of pre- and post-donation GFR in potential living kidney donors
Source: Nephrol Dial Transplant. 2024 Mar 13;39(11):1856–66. doi: 10.1093/ndt/gfae065 (PMC11648953; doi:10.1093/ndt/gfae065)
Supplement: gfae065_Supplemental_File [file gfae065_Supplemental_File.docx]

**Figure S1. Bland-Altman plots of pre-donation CKD-EPI equations and pre-donation mGFR in the total cohort**

Legend: upper = pre-donation eGFR_creat-2009_ with pre-donation mGFR; middle = pre-donation eGFR_cysC-2012_ with pre-donation mGFR; lower = pre-donation eGFR_combined-2021_ with pre-donation mGFR. Bias calculated as eGFR minus mGFR: positive bias indicates overestimation and negative bias represents underestimation. Values outside the 95% confidence interval of the bias are displayed in red.

**Figure S2. Scatter plots of pre-donation CKD-EPI equations and post-donation mGFR in the total cohort**

A

B

C

Legend: All figures: Y-axis = post-donation mGFR. Panel A: X-axis = eGFR_creat-2009_; Panel B: X-axis = eGFR_cysC-2012_; Panel C: X-axis = eGFR_combined-2021_.

**Figure S3. Bland-Altman plots of pre-donation CKD-EPI equations and pre-donation mGFR in donors with high/low muscle mass**

Legend: upper = pre-donation eGFR_creat-2009_ with pre-donation mGFR in donors with high or low muscle mass (N=118); middle = pre-donation eGFR_cysC-2012_ with pre-donation mGFR in donors with high or low muscle mass (N=118); lower = pre-donation eGFR_combined-2021_ with pre-donation mGFR in donors with high or low muscle mass (N=118). Bias calculated as eGFR minus mGFR: positive bias indicates overestimation and negative bias represents underestimation. Values outside the 95% confidence interval of the bias are displayed in red.

**Figure S4. Scatter plots of pre-donation CKD-EPI equations and post-donation mGFR in donors with high/low muscle mass**

A

B

C

Legend: All figures: Y-axis = post-donation mGFR. Panel A: X-axis = eGFR_creat-2009_; Panel B: X-axis = eGFR_cysC-2012_; Panel C: X-axis = eGFR_combined-2021_.

**Table S1. Multivariable linear regression models of the association between pre-donation serum creatinine/cystatin C with pre-donation mGFR**

|  | **St.β** | **R^2^** | **P** | **95% CI** |
| --- | --- | --- | --- | --- |
| *Model 1* |  |  |  |  |
| Age | -0.49 | 0.47 | <0.001 | -0.57 to -0.43 |
| Female sex | -0.42 |  | <0.001 | -0.51 to -0.34 |
| Creatinine | -0.52 |  | <0.001 | -0.61 to -0.44 |
| *Model 2* |  |  |  |  |
| Age | -0.41 | 0.53 | <0.001 | -0.48 to -0.34 |
| Female sex | -0.42 |  | <0.001 | -0.50 to -0.34 |
| Creatinine | -0.40 |  | <0.001 | -0.50 to -0.33 |
| Cystatin C | -0.30 |  | <0.001 | -0.37 to -0.22 |

R^2^ change model 2 vs. model 1: P<0.001

Abbreviations: mGFR: measured glomerular filtration rate.

**Table S2. Multivariable linear regression models of the association between pre-donation serum creatinine/cystatin C with post-donation mGFR**

|  | **St.β** | **R^2^** | **P** | **95% CI** |
| --- | --- | --- | --- | --- |
| *Model 1* |  |  |  |  |
| Age | -0.44 | 0.32 | <0.001 | -0.57 to -0.30 |
| Female sex | -0.26 |  | <0.001 | -0.39 to -0.12 |
| Creatinine | -0.44 |  | <0.001 | -0.55 to -0.34 |
| *Model 2* |  |  |  |  |
| Age | -0.37 | 0.40 | <0.001 | -0.48 to -0.27 |
| Female sex | -0.27 |  | <0.001 | -0.39 to -0.14 |
| Creatinine | -0.32 |  | <0.001 | -0.45 to -0.19 |
| Cystatin C | -0.31 |  | <0.001 | -0.43 to -0.20 |

R^2^ change model 2 vs. model 1: P<0.001

Abbreviations: mGFR: measured glomerular filtration rate.

**Table S3. Characteristics of subgroup with muscle mass (24 hour creatinine excretion) in lowest and highest quartile**

|  | **Lowest quartile** | **Highest quartile** |
| --- | --- | --- |
| **N** | 121 | 122 |
| **Age**, years | 60±11 | 51±11 |
| **Female sex**, n (%) | 61 (50%) | 61 (50%) |
| **Weight**, kg | 75±12 | 86±13 |
| **Height**, cm | 173±10 | 176±9 |
| **BMI**, kg/m^2^ | 25±4 | 28±3 |
| **BSA**, m^2^ | 1.89±0.18 | 2.03±0.19 |
| **Waist to hip-ratio** | 0.91±0.11 | 0.92±0.10 |
| **SBP**, mmHg | 131±15 | 125±15 |
| **Plasma creatinine,** µmol/L | 75±15 | 82±15 |
| **Plasma cystatin C**, mg/L | 0.93±0.14 | 0.86±0.15 |
| **Height-indexed 24 hour creatinine excretion**, mmol/24h per meter | 5.2 [4.5 to 6.8] | 9.6 [7.4 to 10.6] |
| **mGFR**, mL/min/1.73m^2^ | 88±15 | 98±15 |
| **eGFR_creat-2009_**, mL/min/1.73m^2^ | 87±14 | 85±15 |
| **eGFR_cysC-2012_**, mL/min/1.73m^2^ | 84±16 | 95±18 |
| **eGFR_combined-2012_**, mL/min/1.73m^2^ | 86±14 | 91±16 |
| **eGFR_creat-2021_**, mL/min/1.73m^2^ | 91±14 | 88±15 |
| **eGFR_combined-2021_**, mL/min/1.73m^2^ | 91±14 | 95±15 |
| **EKFC_creat_**, mL/min/1.73m^2^ | 82±14 | 82±15 |
| **EKFC_CysC_**, mL/min/1.73m^2^ | 77±15 | 88±16 |
| **EKFC_combined_**, mL/min/1.73m^2^ | 80±13 | 85±14 |

Normally distributed variables: mean±SD, not normally distributed variables: median [IQR], binary variables: N (%).

Abbreviations: BMI: body mass index; BSA: body surface area; creat: creatinine; cysC: cystatin C; eGFR: estimated glomerular filtration rate; EKFC: European Kidney Function Consortium; mGFR: measured glomerular filtration rate; SBP: systolic blood pressure.

**Table S4. Characteristics of subgroups according to cystatin C assay**

|  | **Roche** | **Gentian** |
| --- | --- | --- |
| **N** | 146 | 340 |
| **Age**, years | 56±12 | 56±11 |
| **Female sex**, n (%) | 76 (52%) | 185 (54%) |
| **Weight**, kg | 79±13 | 80±14 |
| **Height**, cm | 174±10 | 174±9 |
| **BMI**, kg/m^2^ | 26±3 | 26±4 |
| **BSA**, m^2^ | 1.93±0.19 | 1.95±0.20 |
| **Waist to hip-ratio** | 0.92±0.10 | 0.90±0.10 |
| **SBP**, mmHg | 125±13 | 126±14 |
| **Plasma creatinine,** µmol/L | 75±13 | 78±15 |
| **Plasma cystatin C**, mg/L | 0.95±0.13 | 0.86±0.15 |
| **mGFR**, mL/min/1.73m^2^ | 95±16 | 93±15 |
| **eGFR_creat-2009_**, mL/min/1.73m^2^ | 89±14 | 86±15 |
| **eGFR_cysC-2012_**, mL/min/1.73m^2^ | 83±15 | 93±17 |
| **eGFR_combined-2012_**, mL/min/1.73m^2^ | 86±14 | 91±15 |
| **eGFR_creat-2021_**, mL/min/1.73m^2^ | 92±14 | 89±14 |
| **eGFR_combined-2021_**, mL/min/1.73m^2^ | 91±14 | 95±15 |
| **EKFC_creat_**, mL/min/1.73m^2^ | 84±14 | 82±14 |
| **EKFC_CysC_**, mL/min/1.73m^2^ | 78±14 | 85±15 |
| **EKFC_combined_**, mL/min/1.73m^2^ | 81±12 | 83±13 |

Normally distributed variables: mean±SD, not normally distributed variables: median [IQR], binary variables: N (%).

Abbreviations: BMI: body mass index; BSA: body surface area; creat: creatinine; cysC: cystatin C; eGFR: estimated glomerular filtration rate; EKFC: European Kidney Function Consortium; mGFR: measured glomerular filtration rate; SBP: systolic blood pressure.

**Table S5. Univariable linear regression analyses of pre-donation serum creatinine/cystatin C and clinical characteristics with pre-donation mGFR in subgroups according to cystatin C assay**

|  | **Roche** | | | **Gentian** | | |
| --- | --- | --- | --- | --- | --- | --- |
|  | **St.β** | **R^2^** | **P** | **St.β** | **R^2^** | **P** |
| **mGFR** | - | - | - | - | - | - |
| **EKFC_creat_** | 0.67 | 0.45 | <0.001 | 0.68 | 0.46 | <0.001 |
| **EKFC_CysC_** | 0.66 | 0.44 | <0.001 | 0.62 | 0.38 | <0.001 |
| **EKFC_combined_** | 0.74 | 0.54 | <0.001 | 0.72 | 0.51 | <0.001 |
| **eGFR_combined-2021_** | 0.71 | 0.49 | <0.001 | 0.71 | 0.50 | <0.001 |
| **eGFR_creat-2021_** | 0.64 | 0.40 | 0.01 | 0.64 | 0.41 | <0.001 |
| **eGFR_combined-2012_** | 0.72 | 0.52 | <0.001 | 0.72 | 0.52 | <0.001 |
| **eGFR_cysC-2012_** | 0.66 | 0.43 | <0.001 | 0.63 | 0.39 | 0.001 |
| **eGFR_creat-2009_** | 0.64 | 0.41 | 0.003 | 0.66 | 0.43 | <0.001 |
| **Plasma cystatin C** | -0.53 | 0.28 | <0.001 | -0.54 | 0.29 | <0.001 |
| **Plasma creatinine** | -0.23 | 0.04 | 0.01 | -0.34 | 0.11 | <0.001 |

Abbreviations: creat: creatinine; CI: confidence interval; cysC: cystatin C; eGFR: estimated glomerular filtration rate; EKFC: European Kidney Function Consortium; mGFR: measured glomerular filtration rate

**Table S6. Donor characteristics of stratified by sex**

|  | **Females** | **Males** |
| --- | --- | --- |
| **N** | 261 | 225 |
| **Age**, years | 56±11 | 56±12 |
| **Female sex**, n (%) | 261 (100%) | 225 (100%) |
| **Weight**, kg | 74±11 | 87±12 |
| **Height**, cm | 168±7 | 181±7 |
| **BMI**, kg/m^2^ | 26±4 | 26±3 |
| **BSA**, m^2^ | 1.83±0.15 | 2.07±0.15 |
| **Waist to hip-ratio** | 0.86±0.10 | 0.96±0.08 |
| **SBP**, mmHg | 124±15 | 128±13 |
| **Plasma creatinine,** µmol/L | 69±10 | 86±13 |
| **Plasma cystatin C**, mg/L | 0.86±0.10 | 0.92±0.15 |
| **Height-indexed 24 hour creatinine excretion**, mmol/24h per meter | 6.0 [5.2 to 6.8] | 8.7 [7.6 to 9.8] |
| **mGFR**, mL/min/1.73m^2^ | 92±16 | 96±16 |
| **eGFR_creat-2009_**, mL/min/1.73m^2^ | 86±14 | 88±16 |
| **eGFR_cysC-2012_**, mL/min/1.73m^2^ | 91±16 | 90±18 |
| **eGFR_combined-2012_**, mL/min/1.73m^2^ | 89±14 | 91±15 |
| **eGFR_creat-2021_**, mL/min/1.73m^2^ | 89±13 | 91±15 |
| **eGFR_combined-2021_**, mL/min/1.73m^2^ | 93±14 | 94±15 |
| **EKFC_creat_**, mL/min/1.73m^2^ | 81±13 | 84±15 |
| **EKFC_CysC_**, mL/min/1.73m^2^ | 86±15 | 80±15 |
| **EKFC_combined_**, mL/min/1.73m^2^ | 83±13 | 82±13 |

Normally distributed variables: mean±SD, not normally distributed variables: median [IQR], binary variables: N (%).

Abbreviations: BMI: body mass index; BSA: body surface area; creat: creatinine; cysC: cystatin C; eGFR: estimated glomerular filtration rate; EKFC: European Kidney Function Consortium; mGFR: measured glomerular filtration rate; SBP: systolic blood pressure.

**Table S7. Univariable linear regression analyses of pre-donation plasma creatinine/cystatin C and clinical characteristics with pre- and post-donation mGFR stratified by sex**

|  | **Pre-donation** | | | | | |
| --- | --- | --- | --- | --- | --- | --- |
|  | **Females (N=261)** | | | **Males (N=225)** | | |
| **Pre-donation** | **St.β [95% CI]** | **R^2^** | **P** | **St.β [95% CI]** | **R^2^** | **P** |
| **mGFR** | - | - | - | - | - | - |
| **EKFC_creat_** | 0.68 [0.62 – 0.80] | 0.46 | <0.001 | 0.66 [0.54 – 0.72] | 0.44 | <0.001 |
| **EKFC_CysC_** | 0.60 [0.52 – 0.72] | 0.36 | <0.001 | 0.67 [0.56 – 0.76] | 0.45 | <0.001 |
| **EKFC_combined_** | 0.71 [0.64 – 0.82] | 0.51 | <0.001 | 0.74 [0.62 – 0.79] | 0.55 | <0.001 |
| **eGFR_combined-2021_** | 0.68 [0.59 – 0.77] | 0.45 | <0.001 | 0.71 [0.61 – 0.80] | 0.50 | <0.001 |
| **eGFR_creat-2021_** | 0.65 [0.56 – 0.74] | 0.42 | <0.001 | 0.63 [0.52 – 0.73] | 0.40 | <0.001 |
| **eGFR_combined-2012_** | 0.70 [0.62 – 0.79] | 0.49 | <0.001 | 0.71 [0.61 – 0.80] | 0.50 | <0.001 |
| **eGFR_CysC-2012_** | 0.57 [0.47 – 0.68] | 0.32 | <0.001 | 0.64 [0.53 – 0.73] | 0.40 | <0.001 |
| **eGFR_creat-2009_** | 0.68 [0.58 – 0.77] | 0.45 | <0.001 | 0.62 [0.51 – 0.72] | 0.39 | <0.001 |
| **Plasma cystatin C** | -0.49 [-0.60 - -0.39] | 0.24 | <0.001 | -0.60 [-0.70 - -0.49] | 0.36 | <0.001 |
| **Plasma creatinine** | -0.46 [-0.57 - -0.35] | 0.21 | <0.001 | -0.49 [-0.60 - -0.37] | 0.23 | <0.001 |
|  | **Post-donation** | | | | | |
|  | **Females (N=120)** | | | **Males (N=116)** | | |
| **Pre-donation** | **St.β [95% CI]** | **R^2^** | **P** | **St.β [95% CI]** | **R^2^** | **P** |
| **mGFR** | 0.77 [0.66 -0.90] | 0.59 | <0.001 | 0.78 [0.66 – 0 .90] | 0.61 | <0.001 |
| **EKFC_creat_** | 0.57 [0.45 – 0.77] | 0.32 | <0.001 | 0.55 [0.38 – 0.67] | 0.30 | <0.001 |
| **EKFC_CysC_** | 0.49 [0.36 – 0.71] | 0.23 | <0.001 | 0.63 [0.49 – 0.77] | 0.40 | <0.001 |
| **EKFC_combined_** | 0.59 [0.46 – 0.77] | 0.34 | <0.001 | 0.68 [0.52 – 0.79] | 0.45 | <0.001 |
| **eGFR_combined-2021_** | 0.53 [0.37 – 0.68] | 0.27 | <0.001 | 0.66 [0.53 – 0.80] | 0.44 | <0.001 |
| **eGFR_creat-2021_** | 0.53 [0.37 – 0.68] | 0.27 | <0.001 | 0.52 [0.37 – 0.68] | 0.27 | <0.001 |
| **eGFR_combined-2012_** | 0.57 [0.42 – 0.72] | 0.32 | <0.001 | 0.68 [0.55 – 0.82] | 0.46 | <0.001 |
| **eGFR_CysC-2012_** | 0.43 [0.26 – 0.59] | 0.18 | <0.001 | 0.62 [0.47 – 0.76] | 0.38 | <0.001 |
| **eGFR_creat-2009_** | 0.58 [0.43 – 0.73] | 0.33 | <0.001 | 0.55 [0.40 – 0.71] | 0.30 | <0.001 |
| **Plasma cystatin C** | -0.33 [-0.50 - -0.16] | 0.10 | <0.001 | -0.59 [-0.74 - -0.43] | 0.34 | <0.001 |
| **Plasma creatinine** | -0.39 [-0.56 - -0.23] | 0.15 | <0.001 | -0.36 [-0.53 - -0.19] | 0.12 | <0.001 |

*For both outcomes in the total cohort, P-values of all associations were <0.001.

Abbreviations: creat: creatinine; CI: confidence interval; CysC: cystatin C; eGFR: estimated glomerular filtration rate; EKFC: European Kidney Function Consortium; mGFR: measured glomerular filtration rate

**Table S8. Accuracy and precision of the eGFR equations for pre-donation mGFR stratified by sex**

|  | **Accuracy and precision pre-donation eGFR for pre-donation mGFR in females (N=261)** | | | | | | | |
| --- | --- | --- | --- | --- | --- | --- | --- | --- |
|  | **eGFR_creat-2009_** | **eGFR_CysC-2012_** | **eGFR_combined-2012_** | **eGFR_creat-2021_** | **eGFR_combined-2021_** | **EKFC_creat_** | **EKFC_CysC_** | **EKFC_combined_** |
| **R squared** | 0.45 | 0.32 | 0.49 | 0.42 | 0.45 | 0.46 | 0.36 | 0.51 |
| **Bias [95% CI]** | -6.5 [-7.8 to -5.0] | -1.6 [-3.4 to 0.24] | -3.0 [-4.4 to -1.5] | -2.9 [-4.4 to -1.4] | 1.4 [-0.1 to 2.9] | -11.0 [-12.4 to -9.5] | -6.4 [-8.0 to 4.7] | -8.7 [-10.0 to -7.3] |
| **RMSE** | 11.9 | 14.7 | 11.5 | 12.2 | 11.9 | 11.7 | 13.5 | 11.0 |
| **IQR bias** | -13.7 to 0.7 | -11.2 to 10.3 | -10.3 to 4.8 | -11.1 to 4.6 | -7.2 to 10.2 | -18.6 to -3.4 | -15.7 to -3.8 | -15.8 to -1.3 |
| **P_30_ [95% CI]** | 97.2% [94.4 to 98.8] | 95.3% [91.8 to 97.4] | 99.2% [97.5 to 99.8] | 97.6% [94.8 to 99.0] | 98.0% [95.3 to 99.3] | 95.7% [92.3 to 97.7] | 95.3% [91.8 to 97.4] | 98.4% [95.9 to 99.5] |
| **P_10_ [95% CI]** | 51.6% [45.5 to 57.7] | 40.9% [35.1 to 47.1] | 57.5% [51.3 to 63.4] | 58.7% [52.5 to 64.5] | 53.5% [47.4 to 59.6] | 40.2% [34.3 to 46.3] | 46.1% [40.0 to 52.2] | 44.5% [38.5 to 50.6] |
|  | **Accuracy and precision pre-donation eGFR for pre-donation mGFR in males (N=225)** | | | | | | | |
| **R squared** | 0.39 | 0.40 | 0.50 | 0.40 | 0.50 | 0.44 | 0.45 | 0.55 |
| **Bias [95% CI]** | -7.4 [-9.2 to 5.6] | -5.7 [-7.6 to -3.7] | -5.7 [-7.2 to -4.1] | -4.1 [-5.8 to -2.3] | -1.6 [-3.2 to -0.01] | -11.8 [-13.5 to -10.2] | -15.6 [-17.2 to -13.9] | -13.7 [-15.1 to -12.3] |
| **RMSE** | 13.6 | 14.7 | 11.9 | 13.1 | 11.8 | 12.4 | 12.7 | 10.7 |
| **IQR bias** | -17.2 to 1.6 | -15.3 to 3.0 | -14.4 to 1.6 | -13.4 to 5.0 | -10.4 to 6.7 | -20.7 to -3.6 | -24.0 to -6.3 | -21.1 to -5.6 |
| **P_30_ [95% CI]** | 95.9% [92.3 to 97.9] | 92.7% [88.4 to 95.5] | 98.6% [95.9 to 99.7] | 9.3% [94.0 to 98.9] | 97.7% [94.6 to 99.2] | 94.1% [90.0 to 96.6] | 87.2% [82.1 to 91.0] | 97.7% [94.6 to 99.2] |
| **P_10_ [95% CI]** | 46.1% [39.6 to 52.7] | 48.4% [41.9 to 55.0] | 52.5% [45.9 to 59.0] | 49.3% [42.8 to 55.9] | 55.7% [49.1 to 62.1] | 37.0% [30.9 to 43.6] | 27.4% [21.9 to 33.7] | 32.4% [26.6 to 38.9] |

Bias calculated as eGFR minus mGFR: positive bias represents overestimation and negative bias represents underestimation.

Abbreviations: eGFR: estimated glomerular filtration rate; EKFC: European Kidney Function Consortium; IQR: interquartile range; mGFR: measured glomerular filtration rate; P_30_ and P_10_: percentage of bias within 30 or 10% of mGFR; RMSE: root mean squared error.

**Table S9. Accuracy and precision of the eGFR equations for post-donation mGFR**

|  | **Accuracy and precision pre-donation eGFR for post-donation mGFR in females (N=120)** | | | | | | | |
| --- | --- | --- | --- | --- | --- | --- | --- | --- |
|  | **eGFR_creat-2009_** | **eGFR_CysC-2012_** | **eGFR_combined-2012_** | **eGFR_creat-2021_** | **eGFR_combined-2021_** | **EKFC_creat_** | **EKFC_CysC_** | **EKFC_combined_** |
| **R squared** | 0.33 | 0.18 | 0.32 | 0.27 | 0.27 | 0.32 | 0.23 | 0.34 |
| **Bias [95% CI]** | -4.5 [-6.0 to -3.1] | -0.2 [-2.0 to 1.7] | -1.4 [-3.0 to 0.1] | -2.8 [-4.3 to -1.2] | -1.2 [-0.4 to 2.7] | -8.0 [-9.5 to -6.6] | -3.8 [-5.4 to -2.1] | -5.9 [-7.3 to -4.4] |
| **RMSE** | 8.2 | 10.2 | 8.4 | 8.6 | 8.7 | 8.2 | 9.1 | 8.0 |
| **IQR bias** | -10.6 to 0.7 | -6.8 to 6.3 | -7.3 to 3.8 | -8.4 to 2.4 | -4.7 to 6.5 | -13.7 to -2.5 | -9.6 to 1.6 | -11.6 to -1.6 |
| **P_30_ [95% CI]** | 97.5% [92.6 to 99.5] | 92.5% [86.2 to 96.2] | 95.0% [89.3 to 97.9] | 98.3% [93.8 to 99.9] | 94.2% [88.2 to 97.4] | 93.3% [87.2 to 96.8] | 94.2% [88.2 to 97.4] | 96.7% [91.5 to 99.0] |
| **P_10_ [95% CI]** | 49.2% [40.4 to 58.0] | 47.5% [38.8 to 56.4] | 57.5% [48.6 to 66.0] | 50.8% [42.0 to 59.6] | 53.3% [44.4 to 62.0] | 35.0% [27.0 to 43.9] | 48.3% [39.6 to 57.2] | 46.7% [38.0 to 55.6] |
|  | **Accuracy and precision pre-donation eGFR for post-donation mGFR in males (N=116)** | | | | | | | |
| **R squared** | 0.30 | 0.38 | 0.46 | 0.27 | 0.44 | 0.30 | 0.40 | 0.45 |
| **Bias [95% CI]** | -2.5 [-4.3 to -0.7] | -0.8 [-2.6 to 1.0] | -1.0 [-2.4 to 0.5] | -1.3 [-3.1 to 0.5] | 1.4 [-0.2 to 2.9] | -6.6 [-8.3 to -4.9] | -8.0 [-9.6 to -6.4] | -7.3 [-8.7 to -5.9] |
| **RMSE** | 9.8 | 9.8 | 8.1 | 9.7 | 8.2 | 9.2 | 8.6 | 7.7 |
| **IQR bias** | -6.6 to 4.3 | -7.7 to 4.7 | -5.2 to 4.5 | -6.9 to 6.1 | -3.5 to 6.6 | -11.7 to -0.5 | -13.7 to -2.7 | -12.0 to -2.1 |
| **P_30_ [95% CI]** | 93.1% [86.8 to 96.7] | 93.1% [86.8 to 96.7] | 98.3% [93.5 to 99.9] | 94.8% [88.9 to 97.8] | 98.3% [93.5 to 99.9] | 93.1% [86.8 to 96.7] | 91.4% [84.7 to 95.4] | 96.6% [91.2 to 98.9] |
| **P_10_ [95% CI]** | 52.6% [43.6 to 61.4] | 50.0% [41.0 to 59.0] | 64.7% [55.6 to 72.8] | 48.3% [39.4 to 57.3] | 53.4% [44.4 to 62.3] | 44.8% [36.1 to 53.9] | 31.9% [24.1 to 40.9] | 43.1% [34.5 to 52.2] |

For calculation of the bias of pre-donation eGFR for post-donation mGFR, we first calculated the predicted post-donation mGFR value by multiplying pre-donation eGFR by 0.66. The bias was then calculated as the difference between predicted post-donation mGFR (0.66*pre-donation eGFR) and true mGFR: positive bias represents overestimation and negative bias represents underestimation.

Abbreviations: eGFR: estimated glomerular filtration rate; EKFC: European Kidney Function Consortium; IQR: interquartile range; mGFR: measured glomerular filtration rate; P_30_ and P_10_: percentage of bias within 30 or 10% of mGFR; RMSE: root mean squared error.
